# Excess degassing drives long-term volcanic unrest at Nevado del Ruiz

**DOI:** 10.1038/s41598-024-51380-5

**Published:** 2024-01-12

**Authors:** João Lages, Zoraida Chacón, Julian Ramirez, Alessandro Aiuppa, Santiago Arellano, Marcello Bitetto, Julián O. Peña, Diego Coppola, Marco Laiolo, Francesco Massimetti, Lina Castaño, Carlos Laverde, Giancarlo Tamburello, Gaetano Giudice, Cristian Lopez

**Affiliations:** 1 Dipartimento DiSTeM, Università Degli Studi Di Palermo, Palermo, Italy; 2 Servicio Geológico Colombiano, Observatorio Vulcanológico y Sismológico de Manizales, Manizales, Colombia; 3 Department of Space, Earth and Environment, Chalmers University of Technology, Gothenburg, Sweden; 4 Dipartimento Di Scienze Della Terra, Università Di Torino, Torino, Italy; 5 Istituto Nazionale Di Geofisica E Vulcanologia, Sezione Di Bologna, Bologna, Italy; 6 Istituto Nazionale Di Geofisica E Vulcanologia, Osservatorio Etneo, Catania, Italy

**Keywords:** Geochemistry, Volcanology

## Abstract

This study combines volcanic gas compositions, SO_2_ flux and satellite thermal data collected at Nevado del Ruiz between 2018 and 2021. We find the Nevado del Ruiz plume to have exhibited relatively steady, high CO_2_ compositions (avg. CO_2_/S_T_ ratios of 5.4 ± 1.9) throughout. Our degassing models support that the CO_2_/S_T_ ratio variability derives from volatile exsolution from andesitic magma stored in the 1–4 km depth range. Separate ascent of CO_2_-rich gas bubbles through shallow (< 1 km depth), viscous, conduit resident magma causes the observed excess degassing. We infer that degassing of ~ 974 mm^3^ of shallow (1–4 km) stored magma has sourced the elevated SO_2_ degassing recorded during 2018–2021 (average flux ~ 1548 t/d). Of this, only < 1 mm^3^ of magma have been erupted through dome extrusion, highlighting a large imbalance between erupted and degassed magma. Escalating deep CO_2_ gas flushing, combined with the disruption of passive degassing, through sudden accumulation and pressurization of bubbles due to lithostatic pressure, may accelerate volcanic unrest and eventually lead to a major eruption.

## Introduction

Volcano monitoring and eruption forecasting have greatly benefited from recent technological advances that allow high temporal resolution measurements of volcanic gas compositions and fluxes. Volcanic gases measured at the surface are the only direct chemical probe of magma at depth and may, by their composition and/or flux, indicate movement of magma toward the surface, changes in the permeability of the shallow conduit system, or pressurization of the magma column beneath a lava dome^1–4^.

Therefore, improving geochemical monitoring infrastructures, and enabling real-time analysis and interpretation protocols, are paramount to our understanding of pre- and syn-eruptive behavior of persistently degassing volcanoes and to mitigate the risk they pose to vulnerable communities.

Nevado del Ruiz, in Colombia, is a 5.321 m-high glacier-clad andesitic volcano in the Cordillera Central of the northern Andes. The volcano erupted numerous times during the Holocene. Its 20th century eruptive history was marked by a period of unrest beginning in late November 1984 with a sharp increase in fumarolic activity^5^. It culminated with an eruption on 13 November 1985, which generated large lahars and killed more than 23,000 people^5–7^.

More recently, deformation was noted in 2007, while seismicity and SO_2_ emission rates started increasing in 2010, with SO_2_ fluxes associated with small eruptions in May and June of that year^8–10^ reaching levels in excess of 20,000 t/d in 2012. In the meantime degassing rates between 2005 and 2015 remained high at Nevado del Ruiz, with satellite data showing an average SO_2_ flux of ∼1,074 t/d^11^ leading up to elevated deformation and two peaks in lava dome extrusion rate: a first short-lived pulse in November 2015 and a second lasting most of 2016. By the beginning of this study, extrusion rates had decreased to 0.02m^3^/s (February 2018). These continued to decline until February 2019, when the dome forming eruption eventually ended^12,13^.

Lava domes are structures that result from the extrusion and accumulation of extremely viscous, quasi solid, lava that are commonly formed at andesitic stratovolcanoes like Nevado del Ruiz. Explosive eruptions at lava domes are thought to be caused by spatial and temporal changes in their permeability and of their ability to exsolve and release volatiles^14,15^. Volcanic gas observations, especially if combined with thermal satellite observations^16^, are thus especially relevant to understanding lava dome activity and behaviour^17,18^. For Nevado del Ruiz, no information on the fluxes of other major volatile species, such as H_2_O and CO_2_, was available until 2017, when the first discontinuous measurements started^19^.

This study reports systematic volcanic gas observations (CO_2_/SO_2_ ratios, CO_2_ and SO_2_ fluxes) taken in 2018–2021, a period of declining dome extrusion rates and negligible deformation. Nonetheless, seismicity, gas and ash emissions remained prevalent throughout this study^13^. Our aim is to present a model of the processes sustaining the persistent degassing, and to identify the mechanisms through which volcanic activity may escalate during periods of prolonged (slow) unrest^20^.

## Results

### Volcanic gas compositions

**Figure 1:**
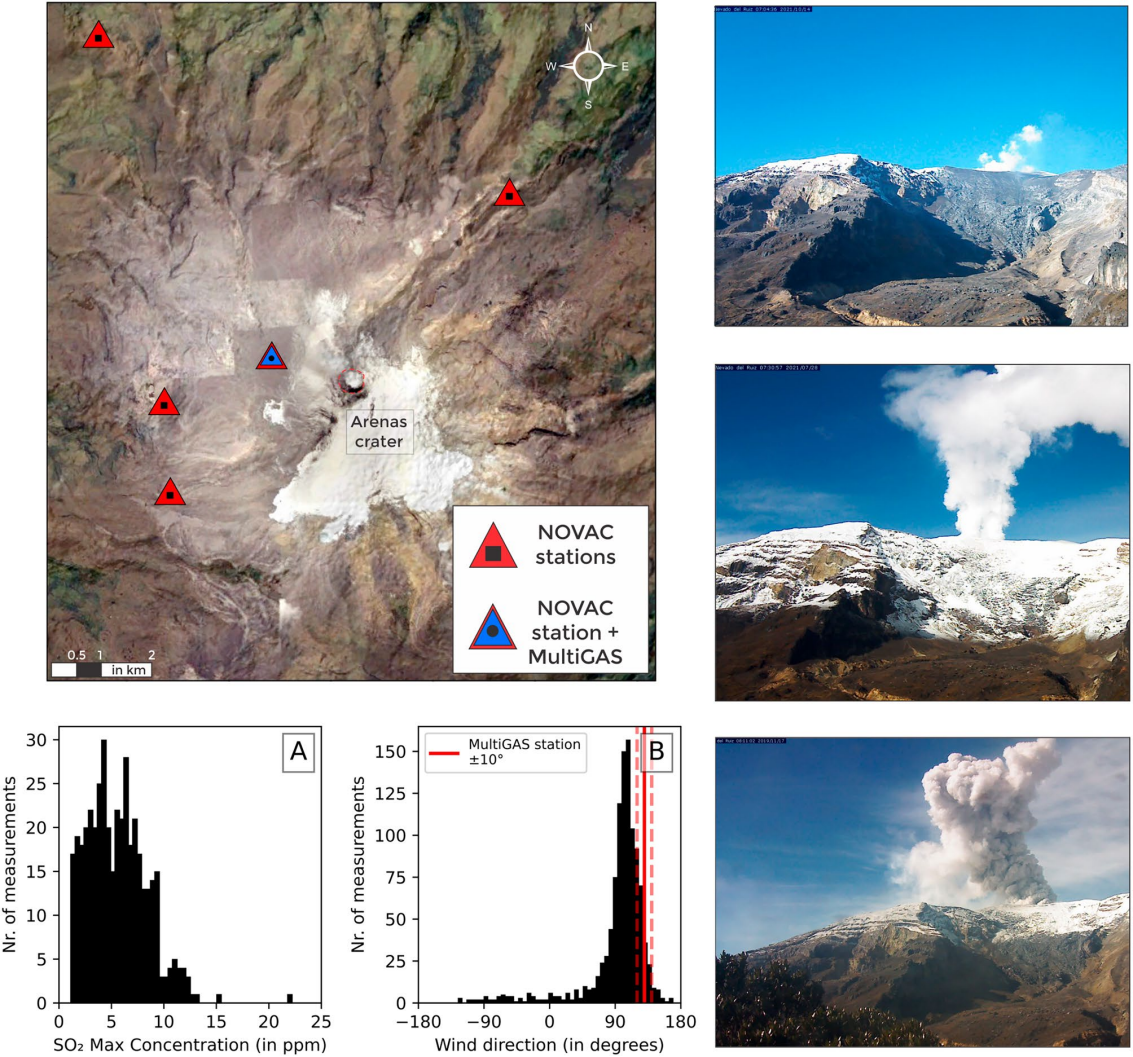
Satellite image of Nevado del Ruiz showing the location of MultiGAS (n = 1) and NOVAC stations (n = 5) used in this study. (**A**) Distribution of SO_2_ Max concentrations recorded by the MultiGAS station between 2018 and 2021; and (**B**) Wind direction data from the NOVAC network, showing good agreement between the location of the permanent MultiGAS station and the predominant wind direction. On the right, photos taken from the monitoring webcams between 2018 and 2021 are courtesy of the *Observatorio Vulcanológico y Sismológico de Manizales (Servicio Geológico Colombiano).*

**Figure 2:**
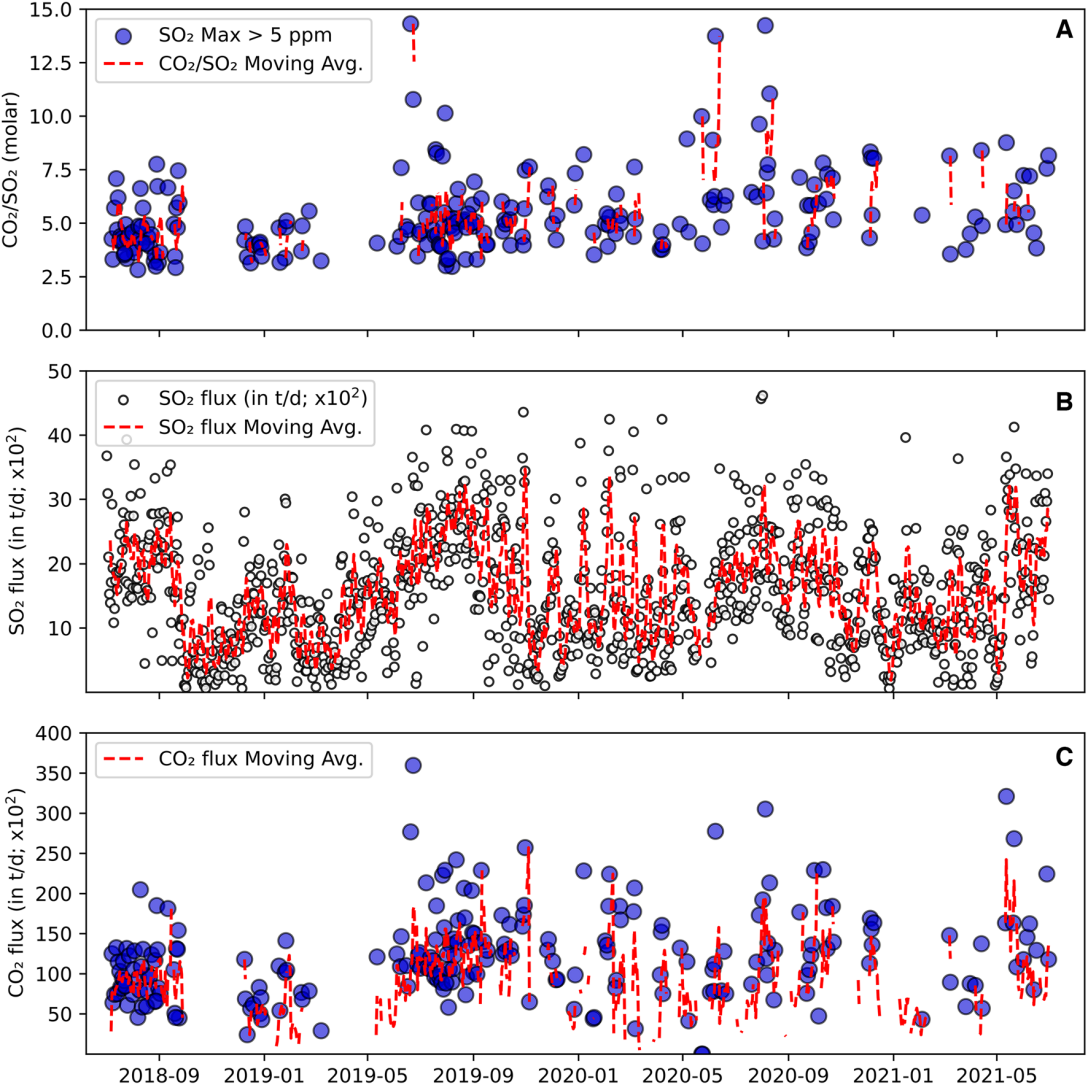
(**A**) CO_2_/SO_2_ compositions (molar); and (**B**) Daily SO_2_ fluxes (averages in t/d; NOVAC Network). (**C**) CO_2_ fluxes are derived from the combination of SO_2_ flux estimates and MultiGAS measurements (see “Methods”). The red lines represent a 10-pt. average (**A**–**C**).

Our results are based on volcanic gas records streamed by a fully autonomous MultiGAS^21,22^ station. The instrument was deployed at Nevado del Ruiz between 2018 and 2021, on the northwest flank of the volcano at an altitude of 4832 m a.s.l. (4.90°N, − 75.34°W; Fig. 1). The data yield^19^ average CO_2_/SO_2_ ratios of 5.4 ± 1.9 (2.8–14.3, n = 220; Fig. 2A; see “Methods”). H_2_S concentrations were rarely detected at > 1 ppm levels, and the H_2_S/SO_2_ ratios are typically <  < 0.1. Volcanic H_2_O signal (above atmospheric background; see “Methods”) is resolved in only 25 acquisitions, due to the very high background (ambient) air H2O concentrations (up to 16,000 ppm) recorded at such altitudes. These yield H_2_O/SO_2_ and H_2_O/CO_2_ ratios averaging at 32.8 (range, 9.1–56.7) and 3.9 (2.6–6.5), respectively. From these measurements, we estimate the average composition of the plume at 84.7 mol% H_2_O, 12.0 mol% CO_2_, 2.8 mol% SO_2_, 0.1 mol% H_2_S, and 0.4 mol% H_2_. Uncertainties in gas ratios measured by the Mulitas are reported in Supplementary Table 1 and are far lower than the variations reported in our time series.

### SO_2_ fluxes

**Figure 3:**
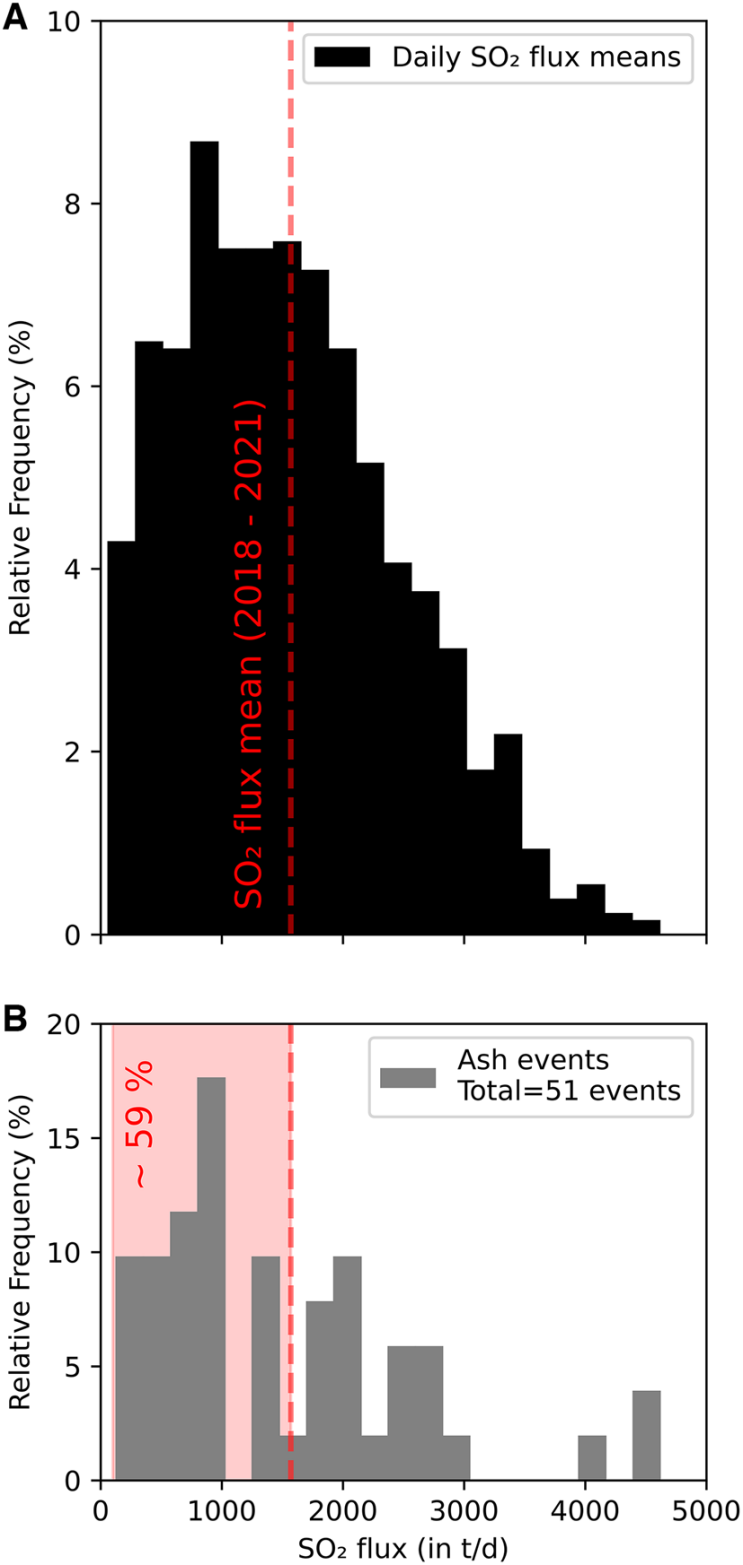
(**A**) Relative frequency distribution of SO_2_ fluxes between 2018–2021 (NOVAC Network; time series shown in Fig. 2B); (**B**) The same data is shown for days in which ash emissions were detected (total of events/days = 51; see “Methods”). Note that in the occurrence of ash emissions approximately 59% of SO_2_ fluxes fall below the 3-year SO_2_ flux average of ~ 1570 t/d.

Daily average SO_2_ fluxes (see “Methods” for data selection criteria and details on daily statistics of SO_2_ emission rates), obtained by the local NOVAC^23^ network of 5 scanning spectrometers between 2018 and 2021, oscillated between 58 and 4617 tons/day, with an average of 1568 tons/day (Fig. 3A). This confirms the sustained degassing activity of Nevado del Ruiz during the investigated time interval. Annual averages show small variations, especially between 2018 (~ 1457 tons/day) and 2019 (~ 1590 tons/day). Four out of the 5 stations yield somewhat similar yearly averages, ranging from ~ 2910 (Bruma) to ~ 4031 t/day (Azufrado/Olleta), thus attesting for the uninterrupted degassing and somewhat unvarying activity at the Arenas crater.

### Volcanic radiative power

**Figure 4:**
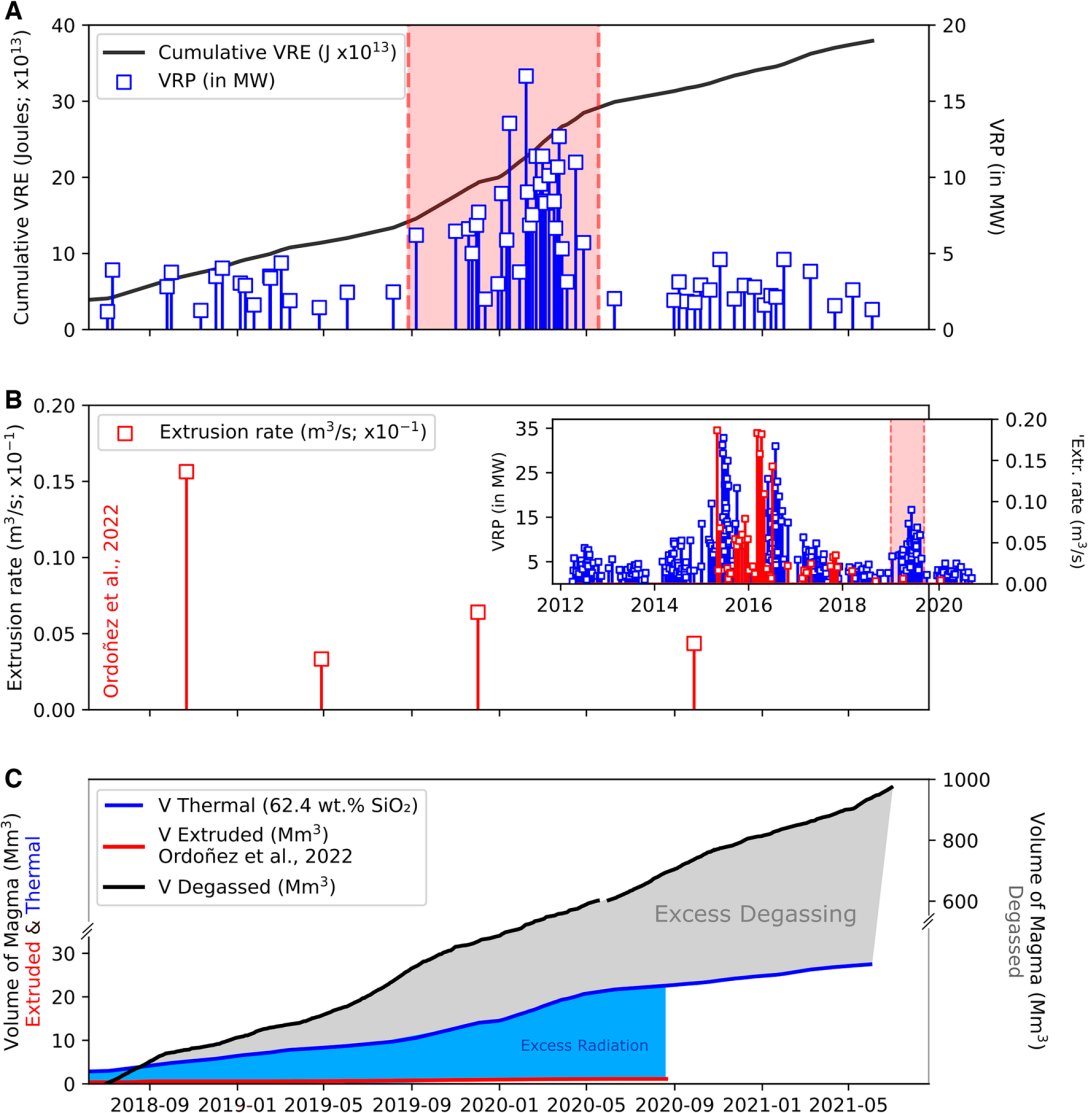
(**A**) Volcanic Radiative Power (VRP; in MW) retrieved from MODIS (blue markers), and associated cumulative thermal energy (Volcanic Radiant Energy; VRE in Joule). High VRP measurements (Jan-Apr 2020) are highlighted by the shaded red area, and also on the inset for comparison with extrusive events of 2015–2016. (**B**) 2018–2021 Extrusion rates reported in Ordoñez et al. (ref^13^ for details). On the inset of B, note the good agreement between VRP data (2012–2021; this study) and extrusion rates, especially for the two extrusion rate peaks detected in November 2015 and for most of 2016^13^. (**C**) Cumulative volumes of degassed (in Mm^3^), thermally radiant (as V_Thermal_) and extruded magma^13^ (see “Methods” for details on calculations).

In the temporal interval investigated, the MIROVA^24^ system detected intermittent thermal anomalies, with a Volcanic Radiative Power (VRP) baseline below 5 MW (Fig. 4A). These relatively low VRP values attest for the overall mild lava extrusion activity registered at Nevado del Ruiz between 2018 and 2021, coupled with continuous high-temperature degassing. Periods of dome extrusion (e.g., Jan–Apr 2020) are clearly detected by MIROVA as VRP maximum values of up to 16.7 MW (see supplementary Table 1–2 for detailed thermal outputs).

## Discussion

### Shallow versus deep magmatic gas signature

The near absence of H_2_S in the gas plume (avg. ∼0.1 mol%) suggests negligible hydrothermal contributions to volcanic gas compositions measured at Nevado del Ruiz between 2018 and 2021. The magmatic nature of the measured gas is additionally supported by the relatively low H_2_O concentrations (maximum 92 mol%). Therefore, we focus on the temporal variations of plume CO_2_/SO_2_ ratios (Fig. 2A), and on the fluctuations of SO_2_ and CO_2_ fluxes (Fig. 2B, C). The in-plume abundances of CO_2_ and SO_2_ both exhibit significant temporal variations. The relatively high CO_2_/SO_2_ ratio range (5.4 ± 1.9) confirms the C-rich nature of Nevado del Ruiz magmatic fluids, interpreted^19,25^ as originating from the recycling of subducted carbonate-rich sediments in the region^26^ (see Aiuppa et al., 2017 for detailed assessment of the relationship between along-arc CO_2_/SO_2_ ratios and subduction sediment compositions). Above average CO_2_/SO_2_ ratios are unlikely to be caused by the scrubbing of volcanic SO_2_ (a process that can cause CO_2_/SO_2_ ratios to exceed typical magmatic values^27^) for two main reasons. Firstly, a typical driver of magmatic S scrubbing is the interaction of deeply ascending magmatic fluids with hydrothermal fluids/ground-water, whereby the conversion of SO_2_ to H_2_S should occur; this is not observed at Nevado del Ruiz, given the negligible amounts of H_2_S measured. Secondly, at andesitic dome-forming volcanoes, SO_2_ scrubbing should be favored in phases when cooling and/or mineral deposition in fractures and pores in the dome carapace^28^ prevail. If this was the case, then high CO_2_/SO_2_ ratios should systematically be associated with reduced SO_2_ fluxes (reduced SO_2_ fluxes have been detected prior to explosion at some dome-forming volcanoes, interpreted as caused by the decreasing in permeability of the main degassing pathways^17^). However, at Nevado del Ruiz, we observe persistently high SO_2_ fluxes (Fig. 2B) that attest to an overall permeable dome, allowing efficient escape of magmatic gases to the atmosphere. We also find no significant correlation between the timing of the summit ash explosions and SO_2_ fluxes (Fig. 3). If we concentrate on the days in which at least one explosion is observed (Fig. 3B), we note that in only 59% of these the daily recorded fluxes are below the 2018–2021 average (41% of the days with explosions recorded higher-than-average SO_2_ fluxes). We caution that we are here interested in long-term (daily to yearly) degassing trends rather than in the driving mechanisms of ash explosions, and we cannot exclude short-term (minutes to tens of minutes) drops in SO_2_ emissivity occur prior to individual explosions (as observed elsewhere^17^) that are not resolvable at the scale of our observations here. In our context, we conclude that clusters of explosions can occur in periods of either reduced (125–1000 t/d) or augmented (2000–3000 t/d, and up to 4617 t/d) daily SO_2_ emission rates. Ultimately, we see no obvious link between compositional changes and shallow processes (scrubbing, dome permeability drop), and we find more likely that the temporally changing CO_2_/SO_2_ ratios are linked to magmatic processes, and potentially to a variable input of deeply rising CO_2_-rich fluids^29^ into the shallow magma plumbing system feeding the dome.

Modelling magmatic degassing requires an understanding of volatile contents in the Nevado de Ruiz parental melts. Stix et al*.,* (ref.^30^) analysed juvenile material erupted at Nevado del Ruiz in November 1985 and September 1989. The authors argued that the wide range of SiO_2_ contents (62.4–76.6 wt%) observed in melt inclusions implies two distinct magmas are at play, one more evolved than the other. This hypothesis is frequently invoked in the post-1985-eruption literature^31–33^. Here we interpret our volcanic gas compositions by using, as proxy for the parental (undegassed) melt composition, the measured volatile contents (2.45 wt% H_2_O and 440 ppm S) in the less evolved (62.4 wt% SiO_2_) melt inclusions^30^. The CO_2_ parental melt concentration has not been characterized at Nevado del Ruiz using melt inclusions. We hence consider a range assumed to be characteristic of initial CO_2_ contents in arc magmas by Plank & Manning, 2019 (1200 ppm, ref.^34^) and Wallace, 2005 (3000 ppm, ref.^35^).

**Figure 5:**
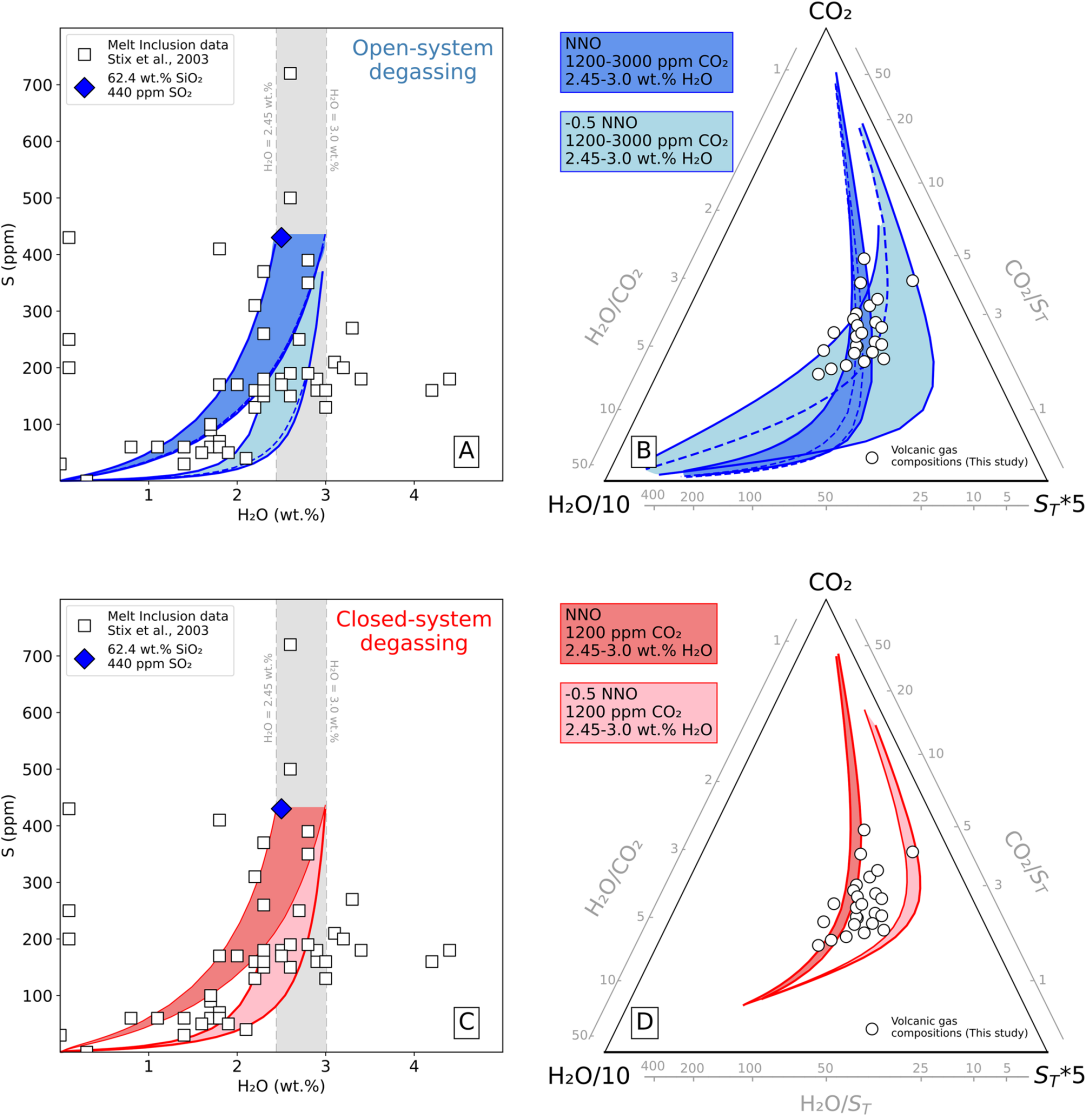
On the left, H_2_O (wt.%) vs S (ppm) in melt inclusions from 1985–1989 eruptive products. Lines illustrate the model-predicted^36^ dissolved H_2_O and S contents in the melt along the modelled (**A**) open- (in blue) and (**C**) closed-system (in red) degassing paths in the 250–0.1 pressure range (see supplementary Table 4 for full input parameters). On the right, triangular plot comparing model-predicted (lines) and measured gas compositions in the H_2_O/10-CO_2_*5-S_T_*10 magmatic system for both open- (**B**) and closed-system (**D**) degassing. Note that model runs fit at large both melt inclusion data^30^ and measured gas compositions.

**Figure 6:**
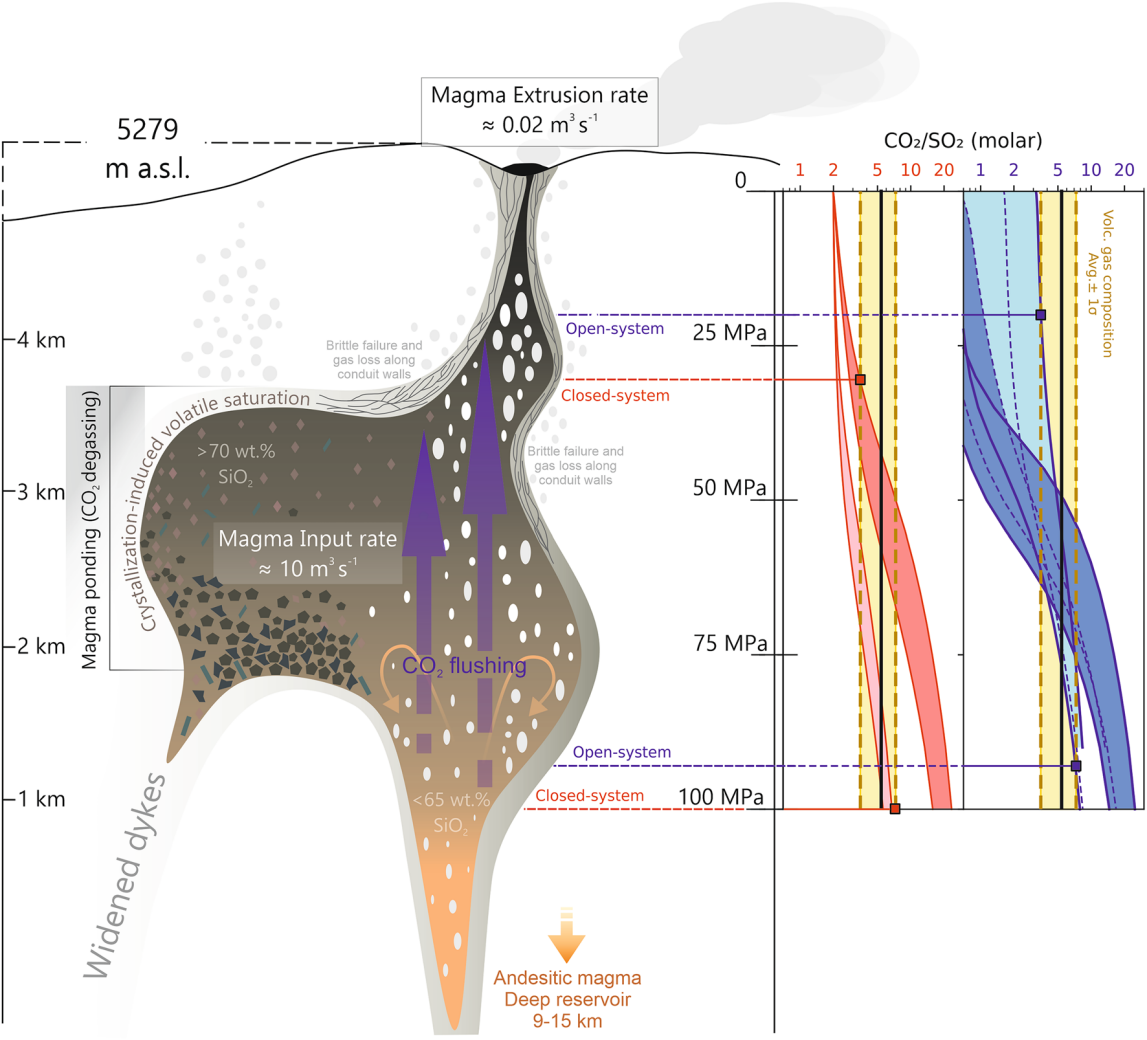
Schematic model of shallow conduit processes in play at Nevado del Ruiz, highlighting the discrepancy between magma input (this study) vs output^13^ rates for the 2018–2021 period. Model-predicted, pressure-dependent evolution of the CO_2_/S_T_ ratio in the magmatic gas coexisting with a Nevado del Ruiz-like melts is shown for the model runs in Fig. 5. Note that the exsolution depths yield by our model runs agree with reservoir depths inferred in the literature^10,38^.

With these initial input parameters, we use a volatile saturation code^36,37^ to calculate the pressure-dependent evolution of the magmatic gas phase exsolved from Nevado del Ruiz magmas upon their ascent and decompression (Figs. 5 and 6). Our simulations are performed in both closed- and open-system conditions (250–0.1 MPa range) at a constant temperature of 900 ^ͦ^ C (1173 K)^32^, and exploring a redox range of 0.5 log units below the nickel-nickel oxide (NNO) buffer (see supplementary Table S4 for detailed input parameters). Note that the large mismatch between degassing and erupted magma volumes (see below) requires gases are separated from melt (e.g., that open system prevails) at some point in the magma ascent/decompression path. However, as the depth/pressure of closed-to-open degassing transition is undetermined, we examine the full closed and full open conditions separately as two end-member scenarios.

Results (Fig. 5) show that the modeled open- and closed-system degassing trends match well the range of gas (this study, Fig. 5B, D) and melt compositions^30^ (Fig. 5A, C) observed at Nevado del Ruiz. We can therefore infer the pressures/depths of gas–melt separation (final equilibration) in the plumbing system by comparing the modeled and observed gas compositions (Fig. 6).

Under closed-system conditions the melt becomes volatile saturated at approximately 250 MPa and our lower/upper range of volcanic gas CO_2_/S_T_ ratios would imply equilibration pressures of approximately 30–100 MPa (~ 1–4 km; Fig. 6). Beyond ~ 30 MPa pressures the magmatic gas phase would evolve to CO_2_/S_T_ compositions lower than those measured in the gas plume (Fig. 6). On the other hand, for the open-system scenario, CO_2_/S_T_ derived pressures/depths range from ∼20 to 93 MPa (∼0.8 to 3.7 km).

Our gas-inferred depth range corresponds to those inferred form melt inclusion entrapment conditions^30^, and to the seismically identified active magma volume^10^. Combined with existing knowledge on the shallow Nevado del Ruiz plumbing system^10,30^, our results identify a main magma storage region in the 1–4 km range, where ponding magma crystallizes (eventually evolving from andesite to dacite), and where gas–melt separation takes place that sustain magmatic gas emissions at the surface. Here, the upper range of our volcanic gas compositions (CO_2_/S_T_ upper range 5.4–7.3; S_T_ stands for total sulfur, and corresponds to the sum SO_2_(g) and H_2_S(g)) may correspond to the roots of such magma storage zone (90–100 MPa pressure; Fig. 6), where separate ascent of deeply-derived CO_2_-rich gas (CO_2_-flushing) starts, eventually followed by separate gas bubble ascent and/or further bubble re-equilibration (1–3 km-depth range). In this interpretation, the shallowest (< 20–40 MPa) portion of the plumbing system would then be occupied by relatively stationary (or poorly mobile), viscous andesitic magma, a very small fraction of which is finally extruded as a dome. In this portion of the reservoir, below-average volcanic gas compositions derive from low-pressure re-equilibration and partial CO_2_ loss from the melt.

Therefore, we argue that the intermittent resupply of the shallow resident conduit magma with more volatile-rich magma (rising from deep) does play a crucial role in sustaining the long-lasting degassing activity of the magmatic column (in addition to causing the brief excursions of gas compositions toward higher CO_2_/S_T_ compositions). In addition, at low confining pressures and high magma viscosities, there may be sufficient strain at the conduit walls to induce brittle failure, with gas loss along permeable channels^38^ (Fig. 6). Such lines of evidence corroborate a multistage model of magma transport and degassing, with alternating periods of magma ascent and ponding^30^.

### Dynamics of shallow ponding conduit magma

Assessments of magma balances (e.g., degassed versus extruded) can provide further constraints on magma feeding processes into the shallow Nevado del Ruiz magmatic system. The volume of degassed magma between 2018 and 2021, inferred from the measured SO_2_ fluxes and knowledge of parental melt S content (see “Methods”), is ~ 974 mm^3^ (Fig. 4C ). Additionally, we estimate a mean MIROVA-derived extrusion rate (TADR; see “Methods) of 0.37 m^3^/s (andesite), which is considerably higher than that (0.02 m^3^/s) reported by Ordoñez et al. (ref^13^) for the 2018–2021 period (Fig. 6). Following the equations provided in Coppola et al., 2013 (ref.^39^; see also “Methods”), we calculate that the thermal output recorded requires surface emplacement (extrusion) of about 27.5 mm^3^ of magma (V_thermal_; Fig. 4C), which is approximately 50 times higher than that of the volume extruded (0.56 mm^3^)^13^ during that period.

In other dome-forming volcanoes (e.g. Sabancaya^16^ and Popocatepetl^18^), V_Thermal_ > V_Extruded_ unbalances have been ascribed to an “excess radiation” process whereby the majority of the thermal anomalies (reported as VRP) were sourced by additional processes other than surface dome extrusion^16,18^. We caution that, at Nevado del Ruiz, the latter may be somewhat underestimated, considering the cycles of dome building and partial destruction (potentially sudden) can be relatively short, and hence difficult to capture with the relatively low temporal resolution measurements reported by Ordoñez et al. Short-lived dome (emplacement/destruction) cycles may, in fact, explain (i) the relatively mild explosive activity of the arenas crater and the lack of a major explosive event since the beginning of the long-lasting unrest; and (ii) the relatively efficient (partial) clearing of the top of the magma column allowing for the conduit to sustain a high level of gas permeability.

In any case, the large unbalance between magma input (10 m^3^/s) and output (extrusion, 0.02 m^3^/s) rates, shown in Fig. 4C and schematically illustrated in Fig. 6, indicates that only about 0.2% of the intruded magma finally reaches the surface. Unbalance between supplied and erupted magma (and the notions of excess degassing and thermal radiation highlighted in our dataset) is typical of open-vent-like-behavior and may indicate that, throughout this study, activity (slow unrest) at Nevado del Ruiz was driven by degassing of unerupted magma (see also ref.^16^).

We have so far established that the existing lava dome at Nevado del Ruiz is connected to deeper reservoirs (e.g., 1–3 km depth^30^) through a gas-permeable volcanic conduit (e.g., ref.^41^). On the other hand, magma supply rate and erupted magma volume suggest that less 1% of the intruded magma reaches the surface (see above). If such significant volumes of degassed magma were to be stored at shallow depths beneath Nevado del Ruiz (i.e., in the upper 2 km), measurable deformation was to be expected. On the contrary, the local *Observatorio Vulcanológico y Sismológico de Manizales* reported no significant anomalies (not to the scale of the volumes of non-erupted magma) between 2018 and 2021.

We, therefore, argue against the possibility that large volumes of magma are being stored at shallow levels within the edifice. Models of convecting magma columns^40^ have been evoked to explain excess degassing and thermal radiation associated with dome-forming activity at andesitic volcanoes^16,18,42^. At Nevado del Ruiz, due to significant degassing-induced crystallization in the shallow part of the conduit (Fig. 6), bimodal flow and magma convection may not occur as efficiently as in low-viscosity mafic systems, especially as magma becomes more evolved and stagnant at shallower levels. During the early stages magma crystallization and bubble formation, some extent of counterflows of ascending (non-degassed) and descending (degassed) magma may coexist in the deep (> 3 km) volcanic conduit, therefore boosting the continuous supply and recycling of deep magmatic fluids between reservoirs (Fig. 6).

In the shallower regions of the conduit, gas–melt separation is likely driven by cooling and crystallization of stagnant, viscous andesitic magma. This process concentrates volatiles in the remaining melt phase and eventually causes them to exsolve into bubbles, which in turn propels the steady degassing behavior and gas compositions observed between 2018 and 2021, and permit large fractions of reservoir volatiles to be released without major eruption. Deeper reservoirs connected to shallower regions by dykes provide occasional inputs of CO_2_-rich magma (CO_2_-flushing) which may disturb normal rates of magma ascent and degassing and cause conduit overflow, resulting in the extrusion events recorded in this study.

### The eruptive cycle of Nevado del Ruiz volcano: clues on the possible activity escalation of a slow and steady system

Periods of enhanced activity, such as higher rates of dome growth or explosive activity, are common at volcanoes such as Nevado del Ruiz. However, our results corroborate that “slow” silicic systems can eventually maintain a steady-state volcanic activity behavior for years, without ever transitioning into a climatic phase^20^. Between 2018 and 2021, this “steady-state” behavior has resulted from a complex but overall “balanced” interplay between inputs of volatile-rich magma, shallow magma crystallization and degassing, and dome extrusion, which has only produced relatively mild explosive activity. Similar slow-unrest systems^20^, of equally evolved magma compositions, such as Popocatépetl^17,18^, in Mexico, and Sabancaya^16^, in Peru show similar longevity in their unrest periods and surface activity. Therefore, a crucial question for these systems, and in particular of Nevado del Ruiz, is how, and over what timescales, volcanic activity can escalate into more voluminous/energetic eruptive events of potential threat to vulnerable communities.

The months preceding Nevado del Ruiz’s catastrophic November 13, 1985 eruption were characterized by minor ash emission events that culminated in a relatively small eruption (Volcanic Explosivity Index, VEI = 3)^5,43^. Juvenile scoria and pumices were erupted^31^ and about 90 kt of SO_2_ released^44^, suggesting that the eruption was in fact magmatic and not phreatic^45^. Giggenbach et al*.* (ref.^46^; see also ref.^47^) reported on an extensive hydrothermal system beneath the volcano, which is manifested today entirely through springs and fumaroles spread throughout the large periphery of the volcano. Our volcanic gas data, however, shows that the present high gas and heat fluxes have most likely boiled off any meteoric water and potentially decoupled the hydrothermal and magmatic systems of Nevado del Ruiz. If Nevado del Ruiz is to sustain its current levels of unrest, the origin and nature of a future major eruptive event is therefore likely to be magmatic.

Given the catastrophic consequences of the November 1985 eruption^6^, we must attempt to correlate the pre-, syn- and post-eruptive observations of the historical event with the current unrest signals. We emphasize two major findings: (i) Banks et al. (ref.^48^) reported no deformation and therefore lack of significant intrusive activity prior to and during the 1985 eruptive period; and (ii) the amount of “new” magmatic material produced during the November 1985 eruption was disproportionally small to account for the large amounts of SO_2_^49^ released then and over subsequent periods (see ref.^43^). Based on our findings, a large degassing excess and a lack of deformation are distinctive features of present-day activity, although no major eruption has yet occurred. Our conceptual model (Fig. 6) accounts for different evolving magmas^30^ at shallow depths, which degas extensively over time. The same magma regions were likely involved as source of the large amounts of pre- and syn-eruptive passive degassing observed from 1985 to 1990 and beyond^43^.

**Figure 7:**
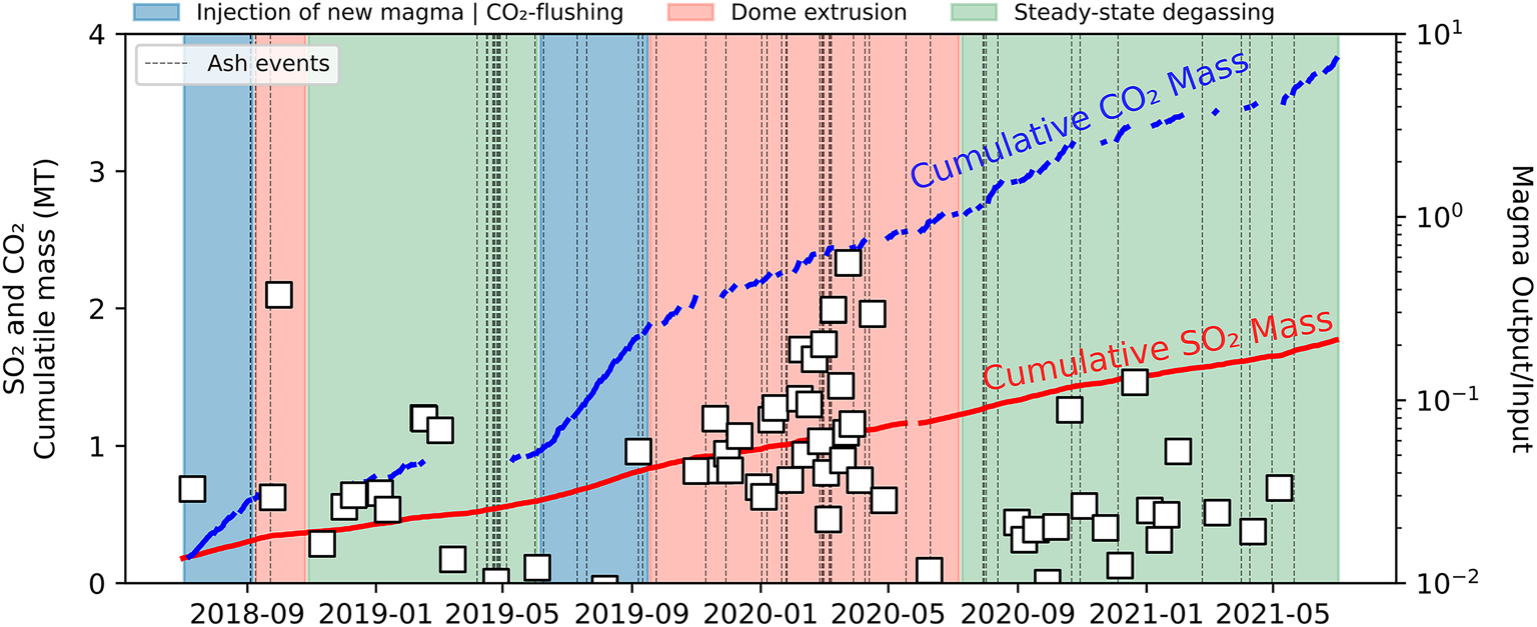
Magma output/input ratio (2018–2021), and CO_2_ and SO_2_ cumulative masses distinguish periods dominated by CO_2_ flushing (deep) and steady-state degassing, with occasional overflow (minor dome extrusion events) of the magma column. Note that, given the good agreement between extrusion rates^13^and VRP data between 2012 and 2021 (see Fig. 4B), we use here magma output rate as TADR (in m/s; see “Methods”) to identify periods of higher extrusion rates between 2018 and 2021.

The mechanisms of gas/magma transfer within the shallow magma plumbing system, and between the shallow and deep magmatic systems, are difficult to constrain. However, our results suggest that crystallization-induced (evolved magma) and CO_2_-rich gases (from deep) are necessary to explain the range of CO_2_/SO_2_ compositions measured at the surface. Depending on magma physical properties (e.g., viscosity, vesicularity, and percentage of interconnected vesicles), each of them can dominate at specific depths or time^17^. In the current degassing unrest, in particular, phases of enhanced CO_2_ flushing can be detected as periods of escalating CO_2_ surface release (blue shaded areas; Fig. 7). Increased gas flushing may render the shallow ponding magma more buoyant, eventually leading to occasional events of dome extrusion (red shaded areas, as identified from increasing magma output/input ratios; Fig. 7) once the top of the magma column overflows. Carbon dioxide (CO_2_) flushing, in particular, may play a crucial role in governing degassing behavior over time. While at present shallow ponding magma may be sourcing the enhanced degassing rates recorded at Nevado del Ruiz (green shaded areas; Fig. 7), ascent of voluminous CO_2_-rich deep gas amounts in the conduit may eventually cause eruption^50^. Volcanic gas release through permeable conduit walls and dome during times of passive degassing may be disrupted by sudden accumulation and pressurization of bubbles due to lithostatic pressure that tends to compact and close the system^17^. The combination of both processes may culminate in a major eruption.

Therefore, monitoring the composition and mass flux of volcanic gases is critical for fully informed forecasting efforts. However, the challenges of real-time measurements of volcanic gas compositions at volcanoes such as Nevado del Ruiz are exacerbated by extreme low ambient temperature conditions and high level of volcanic activity. Nonetheless, our study attests to the advantages of combining composition, flux and satellite remote sensing measurements to efficiently address the dynamics of shallow magma transfer and extrusion at strongly degassing volcanoes. Moreover, by monitoring the Nevado del Ruiz volcanic degassing behavior over the 3-year period, this study crucially distinguishes several activity phases (e.g., CO_2_ gas flushing, dome extrusion, persistent open-conduit degassing) within the recent unrest cycle of Nevado del Ruiz, while highlighting their specific chemical and thermal patterns to future risk assessment efforts.

## Methods

### Permanent MultiGAS station

During operation, the MultiGAS^21,22^ measured in-plume concentrations of CO_2_, SO_2_ and H_2_S at 1 Hz. The permanent station worked for 4 30-min cycles every day between 2018 and 2021, at 0:00, 6:00, 12:00, 18:00 (UTC time). For details on calibration and sensor range see ref.^51^. Ambient pressure, temperature and relative humidity were also measured, which allowed calculation of in-plume H_2_O concentrations using the Arden Buck equation^52^ (Supplementary Table 3). CO_2_/SO_2_ and H_2_O/SO_2_ ratios (supplementary Tables 1 and 3) correspond to the slope of a best-fit regression line of the concentrations (in ppm) of both species in the selected temporal window (Ratiocalc^53^). Results (Fig. 2) are only reported for temporal windows in which the SO_2_ concentration was above the 5 ppmv threshold, and in which correlations between CO_2_ and SO_2_ and H_2_O and SO_2_ exceed an R^2^ of 0.6. Despite the daily measurement routines, our volcanic gas dataset is limited to days in which wind direction favored the southwest sector of the volcano, where the sector Bruma is located (see Fig. 1). For instance, between 2018 and 2019, 1725 acquisitions (30-min each) were successfully transferred via telemetry from Bruma to OVSM, and subsequently processed at the University of Palermo. Approximately 67% of these acquisitions registered SO_2_ concentrations above instrument noise (> 0.2 ppmv), but only about 23% recorded SO_2_ levels ≥ 5 ppm (the minimum concentration threshold here considered above which the plume is sufficiently “dense” to allow for compositional and CO_2_ flux estimates; Fig. 2). Error are expressed as the standard error of the regression analysis and subsequent error propagation, error on inferred flux propagate error on the SO2 fluxes and gas ratios.

### Daily SO_2_***flux estimates***

Sulfur dioxide emissions from Nevado del Ruiz are measured daily by scanning UV spectrometer systems installed through the Network for the Observation of Volcanic and Atmospheric Change project^23,54^. This network includes 5 different scanning locations, Bruma (4.90; − 75.33, 4878 m a.s.l.), Alfombrales (4.88; − 75.35, 4458 m a.s.l.), Azufrado/Olleta (4.89; -75.35, 4909 m a.s.l.), Inderena/El Camion (4.96; − 75.37, 4016 m a.s.l.) and Recio 3 (4.86; − 75.33, 4665 m a.s.l.; see map of Fig. 1) that provide plume scans at virtually all wind directions. The NOVAC scanning mini-DOAS (differential optical absorption spectroscopy; see ref.^55^) instruments scan the sky continuously during daylight hours to measure the integrated absorption of UV light by SO_2_ in the plume. These are then combined with meteorological information to derive daily statistics of total SO_2_ emissions. Wind speed and direction are acquired from local meteorological models from IDEAM (http://www.ideam.gov.co/). Daily SO_2_ flux estimates are combined here with in tandem CO_2_/SO_2_ gas ratios (converted from molar ratios to mass based on concentration ratios) measured by the permanent MultiGAS station to derive CO_2_ flux budgets between 2018 and 2021. The SO_2_ flux dataset assembled over the years by the OVSM highlights a dependence on wind patterns. Specifically, between May and October, westwards plume directions allow ideal scanning geometries for 4 out of the 5 stations (located on the west flank of the volcano). This ultimately translates into higher estimated fluxes comparing to periods during which the plume may become undetected in more than one scan (due to unfavorable transport directions). We here consider only SO_2_ flux measurement scans with complete coverage of the plume (completeness > 0.8), in order to minimize the effect of wind direction in our daily SO_2_ flux estimates.

### CO_2_ fluxes

We derive daily averaged **CO**_**2**_** fluxes** (in t/d; Fig. 2C)by combining CO_2_/SO_2_ ratios (MultiGAS station) and SO_2_ fluxes (NOVAC network), as: $${\text{CO}}_{2} \,{\text{flux}} = {\raise0.7ex\hbox{${{\text{CO}}_{2} }$} \!\mathord{\left/ {\vphantom {{{\text{CO}}_{2} } {{\text{O}}_{2} }}}\right.\kern-\nulldelimiterspace} \!\lower0.7ex\hbox{${{\text{O}}_{2} }$}} \times {\text{SO}}_{2} \,{\text{flux}}\left( {{\text{t}}\,{\text{d}}^{{ - 1}} } \right)$$.

**Sulfur flux** (in kg/s) is calculated from the following:

$${{\text{S}}}_{\mathrm{flux }}\left(\mathrm{kg }{{\text{s}}}^{-1}\right)= \frac{{\text{M}}({\text{S}})}{{\text{M}}({{\text{SO}}}_{2})} \times (\frac{{{\text{SO}}}_{2 }\mathrm{flux }\left(\mathrm{in t }{{\text{d}}}^{-1}\right) \times 1000}{24 \times 60 \times 60}$$).

### Volcanic radiative power (MODIS)

MIROVA^24^ (Middle InfraRed Observation of Volcanic Activity; www.mirovaweb.it) algorithm allows to detect, locate and quantify volcanic hotspots, measuring the heat flux radiated by hot (> 300 °C) volcanic features (VRP ± 30%, Fig. 4A, inset of Fig. 4B). This approach provides the VRP time series (and its associated Volcanic Radiative Energy, VRE; Fig. 4A) recorded at Nevado del Ruiz between 2018 and 2021 and prior. **Volumes of radiating magma (V thermal**; Fig. 4C) are retrieved from the thermal approach, and are related to the measured radiant energy (VRE)^39^ through:

${V }_{Thermal}=\frac{VRE}{{C}_{rad}}$ , where ${C}_{rad}$ is an empirical coefficient that takes into account the effective rheology of the emplacing lava body^39^. For Nevado del Ruiz we used a silica content of 62.4 wt%^30^, obtaining a ${C}_{rad}$ of 2.1 × 10^6^ J/m^3^.

The **volume of degassed magma (V degassed**.; Fig. 4C) takes into account the measured S flux (see above) and calculations of magma input rates^40,56^. It is obtained from the following equation:

${V}_{degassed }= \frac{S flux \left(in kg {s}^{-1}\right)}{\Delta XS \times \rho m \times \phi }$, where ∆XS is the S volatile loss, derived from the difference between parental melt volatile content (440 ppm in melt inclusion data^30^) and the residual S content in the groundmass (as low as 70 ppm^30^); ϕ is the crystal fraction, assumed for Nevado del Ruiz magmas to be around 40%; and $\rho$ m is the melt density of the magma (2227 kg/m^3(30)^).

Same estimates of **magma input rates** are used in Fig. 7. Instead, given the good agreement between VRP and output rates shown in Fig. 4B (and inset), output rates in Fig. 7 are calculated as time-averaged lava discharge rates (TADR)^39^, by considering the following: $TADR=\frac{VRP}{{C}_{rad}}$.

### Ash events record

Ash emission events were registered by the *Observatorio Vulcanológico y Sismológico de Manizales (Servicio Geológico Colombiano)* through observation of webcam video recordings and reports from local communities.

### Supplementary Information

Supplementary Information.
